# Supplementary material for: Generalized decomposition of multivariate information
Source: PLoS One. 2024 Feb 5;19(2):e0297128. doi: 10.1371/journal.pone.0297128 (PMC10843128; doi:10.1371/journal.pone.0297128)
Supplement: Attachment — Submitted filename: review_2023_10_Generalized_decomposition_of_multivariate_information.pdf [file pone.0297128.s001.pdf]

Supplementary revision material of manuscript number: PONE-D-23-29949

Generalized decomposition of multivariate information

October 27, 2023

1 Summary

Inspired by the work of Williams and Beer (2010), the author derived a decomposition for the Kullback-Leibler divergence (KL-divergence) using elements of the Bayesian interpretation of the local information decomposition. In Section 2, a Bayesian formulation of the KL-divergence is introduced, expressing it as the expected value of the difference between prior and posterior surprisal. The author also revisited the partial entropy decomposition, with a focus on local information derivation, inspired by the work of Finn and Lizier (2018, 2020)). This section lays the foundation for information decomposition. The primary contribution, found in Section 3, is the introduction of the local information decomposition of the KL-divergence, referred to as GID. GID is then applied to various information-theoretic measures within the KL-divergence family, offering new insights, especially into TSE Complexity. The manuscript concludes by discussing the conceptual, theoretical, and potential experimental impacts of GID and outlines future directions.

2 Overall Assessment

This manuscript offers a valuable generalization of information decomposition, allowing for the partitioning of information-theoretic measures within the KL-divergence family. This work lays a strong foundation and holds the potential to provide new tools for a deeper understanding of the information-theoretic measures commonly employed by experimentalists. The author's demonstration, particularly in the case of TSE complexity, underscores the practical value of this research.

The manuscript is enjoyable to read and in principle is well-written, but its clarity could benefit from some minor changes as well as correcting a few minor errors (see minor comments). However, we have two major comments where one needs clarification and the other a potential correction. Additionally, in the Section "Inquisitive Comments" we have

a small conceptual feedback on one point raised by the author (and could benefit their manuscript), but unfortunately we didn't have the time to work it out in detail.

3 Major Comments

Synergy interpretation in TSE complexity. In Section 3.1 paragraph "**Tononi-Sporns-Edelman Complexity**", the author argues that synergy is more akin to the balance of integration and segregation (BIS) than being a kind of global integration. They formulated their argument from the assumption that high TSE is a sign of BIS to conclude that since synergy contributes to high TSE then "synergy is akin to BIS". We found this rather a weak argument since Tononi, Sporns, and Edelman's evidence for a positive correlation of the high value of TSE and BIS is theoretically weak in itself (a hypothesis) and thus extends to the author's argument on synergy. We would appreciate a clarification (as we might be mistaken about the high TSE theoretical evidence of BIS) since we think this is an interesting insight into synergy if the argument is to be cleared as strong enough. Hopefully, the author could integrate their clarification into the manuscript too.

The GID of $I(X_1, X_2 : Y)$. In Section 3.2, the author discusses two different ways to formulate $I(X_1, X_2 : Y)$ as a KL-divergence, each resulting in a different GID. This poses a mystery, as reconciling these two GIDs is not straightforward. We noticed, however, that the first formulation of $I(X_1, X_2 : Y)$ is missing an expectation (**equation (26)**). In "red" we provide the equation from the manuscript and in "blue" our potential correction:

$$I(X_1, X_2 : Y) := D(\mathbb{P}(X_1, X_2 \mid Y) \parallel \mathbb{P}(X_1, X_2))$$

$$I(X_1, X_2 : Y) := \mathbb{E}_Y \left[D(\mathbb{P}(X_1, X_2 \mid Y) \parallel \mathbb{P}(X_1, X_2)) \right] \, .$$

We have noticed that the first formulation appears to be an expectation of a KL-divergence formulation, rather than a direct KL-divergence formulation. This could explain how the 18 terms resulting from the GID of the one and only direct KL-divergence formulation of $I(X_1, X_2 : Y)$ (**equation (30)**) can be aggregated to recover the 4 terms resulting from the GID of the first formulation (**equation (26)**). We kindly request the author's review of equation (26) or an argument in support of its correctness. It's worth noting that our suggestion for reconciliation is speculative, and we acknowledge that we have not had the opportunity to mathematically validate it.

4 Minor Comments

There are a few typo mistakes, notation adjustments, and paragraph restructuring. Below is a list of them where quoted text from the manuscript is written with red and the suggestion with blank black. There are items which are marked as optional. For these items, we leave the liberty for the author to take into consideration.

Page 2 Paragraph "A Note on Notation" (Optional)

The author could add for clarity that $X \perp Y$ stands for X and Y are statistically independent. Since it is reoccurring throughout the manuscript with no introduction.

Page 3 Equation (2)

Rather than writing $Informtion$ which is read as the product of variable I, n, f, and so on. The author can use the command "mathrm" so that information is an operator and appears as such Information. The same applies to $prior$ and $posterior$ in the same equation.

Page 4 Section 2.2 Paragraph 4 Formulizing this notion of "shared entropy" turns out to be trivial

This needs a citation.

Page 5 Paragraph 2 The seminal insight of Williams and Beer was that the set of collections of sources required to decompose $\mathbf{x}$ is constrained to the set of all combination of sources such that no source is a subset of any other:

This needs a citation too.

Page 5

This page could benefit from rewriting. We also suggest adding a figure for the redundancy lattice. Especially since the author refers later to the top half and the bottom half of the redundancy lattice without defining them (they are only clear for Information decomposition experts). This for completeness could be cleared in one figure.

Page 5 Equation (15) $h(\mathbf{x}) = h_\partial^{\mathbf{x}}(\{x_1\}\{x_2\} + h_\partial^{\mathbf{x}}(\{x_1\}) + h_\partial^{\mathbf{x}}(\{x_2\}) + h_\partial^{\mathbf{x}}(\{x_1, x_2\})$

There is a missing parenthesis in the term $h_\partial^{\mathbf{x}}(\{x_1\}\{x_2\}$.

Page 6 Paragraph 5 So, $D_\partial^{P\|Q}(\{1\}\{2\}\{3\}) > 0$, there is more "redundant surprise" in the distribution Q and we would be less surprised if we operated based on distribution P. This difference is the information gain redundantly shared by all three variables.

This sentence could be made clearer. The explanation for the negative values in the following paragraph was better and we recommend following that. Also, if we are not mistake their notation is $D_\partial^{\mathbb{P}\|\mathbb{Q}}$ rather than $D_\partial^{P\|Q}$, please correct it.

Page 6 Paragraph 6 ...rather than if we belive $\mathbb{Q}$ (our posterior).

We think $\mathbb{Q}$ is our prior, no?

Page 7 Equation (21) (Optional) $TC(\mathbf{X}) := D\left(\mathbb{P}\|\mathbb{P}\left(\Pi_{i=1}^{|\mathbf{X}|}X_i\right)\right)$.

Is it more common to write $\mathbb{P}\left(\Pi_{i=1}^{|\mathbf{X}|}X_i\right)$ or $\Pi_{i=1}^{|\mathbf{X}|}\mathbb{P}\left(X_i\right)$ when referring to the marginal probability product.

Page 7 Second to last Paragraph

The author is using $H_\partial^{\mathbb{P}}(\{1\}\{2\}\{3\})$ and $H_\partial^{\mathbb{P}}(\{1,2,T\})$. This creates inconsistency in the notion of the triplet whether it is $(1,2,3)$ or $(1,2,T)$. We suggest that the author sticks to $(1,2,T)$ since it is also used in Table 1. Additionally, this paragraph needs rewriting. Possibly dividing it into steps so that the reader can grasp it more easily.

Page 9 Paragraph 2 The first is that terms of the form $TC_\partial^{123}(\{X_i, X_j\})$ do not appear. This term corresponds to the bivariate mutual information between two elements X_i and X_j (since the total correlation of two variables is the mutual information between them).

This might be nitpicking. Nonetheless, $TC_\partial^{123}(\{X_i, X_j\})$ is an atom and so it is part of the bivariate mutual information but doesn't correspond to it. On the other hand, $TC_\cap^{123}(\{X_i, X_j\})$ is what corresponds to the bivariate mutual information. We could have however misunderstood what the author meant by "correspond" and thus this nitpicking comment. Additionally, $TC_\partial^{123}(\{X_i, X_j\})$ could be written in its simplified notation $\{i, j\}$ so that the reader can easily relate to the equation.

Page 9 Paragraph 2 (Optional)

The author could elaborate that the synergy term is $\{1,2,3\}$ and also give examples of the redundancy terms.

Page 9 Paragraph Tononi-Sporns-Edelman Complexity If the system is entirely integrated (i.e. redundant), then the TSE complexity is low. Similarly, if the system is disintegrated (not really a system at all, just a collection of independent elements), then the TSE is also low.

The author uses the term disintegration to refer to complete segregation. It is better to point out that disintegration is a type of segregation since they pointed out this case in the following paragraphs but called it segregation. This is confusing to the reader.

Page 11 and 12 Section 4

This section could benefit from a division into subsections with meaningful titles. It is hard to read as is. The author jumps from one topic to the other with (pardon my French) a clumsy flow. We think this is the not well-written part of the manuscript and its quality is lower than other sections.

Page 12 Last Paragraph in Section 4 (Optional)

The author talks about the potential impact of the work on cognitive science. It was brought to my attention recently that cognitive scientists avoid using Shannon information due to a lack of semantics which is essential to their research. Since cognitive science is not our field we cannot assess the correctness of this statement, we simply brought it to the attention of the author so that they are aware of some critiques that might sway their way.

5 Inquisitive Comments

Integrated infomation decomposition ΦID. The integrated information decomposition partitions the mutual information as follows:

$$I(X_1, X_2 : Y_1, Y_2) = D(\mathbb{P}(X_1, X_2, T_1, T_2) \| \mathbb{P}(X_1, X_2)\mathbb{P}(T_1, T_2)) \ .$$

When performing a GID on $I(X_1, X_2 : Y_1, Y_2)$, it results in 166 atoms. To reconcile these 166 atoms with the 16 atoms of ΦID, one potential approach might involve using the expectation KL-divergence of $I(X_1, X_2 : Y_1, Y_2)$. This would entail considering expectations on the variables Y_1, Y_2, and (Y_1, Y_2) for the forward PID, and X_1, X_2, and (X_1, X_2) for the backward PID. While we haven't worked out the mathematical details, we believe this approach holds promise for addressing their conjecture.

References

C. Finn and J. Lizier. Probability mass exclusions and the directed components of mutual information. *Entropy*, 20(11):826, 2018.

C. Finn and J. T. Lizier. Generalised measures of multivariate information content. *Entropy*, 22(2):216, 2020.

P. L. Williams and R. D. Beer. Nonnegative decomposition of multivariate information. *arXiv preprint arXiv:1004.2515*, 2010.
