# Supplementary material for: Generalized decomposition of multivariate information
Source: PLoS One. 2024 Feb 5;19(2):e0297128. doi: 10.1371/journal.pone.0297128 (PMC10843128; doi:10.1371/journal.pone.0297128)
Supplement: Attachment — Submitted filename: response_to_reviewers.pdf [file pone.0297128.s002.pdf]

Response to Reviewers
Thomas F. Varley, PhD
December 7, 2023

I would like to thank the editor and peer reviewers for their thorough and insightful comments (and kind words). We will respond point-by-point below. Our responses are indicated in blue font.

Reviewer 1

* Could maybe extend this a bit to expand on the PED and give some more examples of the different measures (not at all necessary, just may make the paper useful to a wider range of readers).

We have expanded our discussion of the PED by adding the following discussion making the link between the PID and PED clear:

> Another way that the PED can be understood is as a special case of the partial information decomposition (PID). As discussed above, typically, the PID decomposes the mutual information that some set of variables disclose about a shared target: $I(X_1, \ldots, X_k; T)$. However, the PID can induce a PED in the particular case where the target is the joint state of all the inputs. If $\mathbf{X} = \{X_1, \ldots, X_k\}$, then $I(X_1, \ldots, X_k; \mathbf{X}) = H(\mathbf{X})$, and a PID of the mutual information will decompose the joint entropy of the whole $\mathbf{X}$ (this formulation of the PED was first noted by Makkeh et al., {makkeh_introducing_2021} and then later explored in detail by Varley et al., {varley_partial_2023}). Intuitively, the PED can be understood as decomposing the information that the *parts* disclose about the *whole*.

With respect to the different possible measures, I don't want to get into too much gory detail here (I am kind of assuming that any reader who is moved to read this paper in detail is *probably* already familiar with PID). However, I have added the following short discussion:

> Since the original introduction of the PID, various teams have proposed a plethora of redundancy functions that satisfy the Williams and Beer axioms and consequently induce the redundancy lattice. These different redundancy functions can return very different results making the problem of picking the "right" function a tricky one. For a partial review, see {kolchinsky_novel_2022}.
>
> ...
>
> Given the relationship between the PID and PED described above, in theory, any redundant information function could be used to induce a PED, however, the generalized information decomposition imposes additional constraints. The most significant is that the redundancy function must be localizable (i.e. the $I_{\min}$ function proposed by Williams and Beer will not work, as it is not fully localizable). All three existing redundant entropy functions (h_{cs} {ince_partial_2017}, $h_{\min}$ {finn_generalised_2020} and h_{sx} {varley_partial_2023}) satisfy this property. Additionally, it would be helpful to require that the local partial entropy atoms be strictly non-negative. This rules out one of the three functions: h_{cs} {ince_partial_2017}, being based on the local co-information, can return negative partial entropy atoms, which significantly complicates the interpretation of the PED and the GID (discussed below). The two remaining functions, $h_{\min}$ {finn_generalised_2020} and h_{sx} {varley_partial_2023} do return strictly non-negative partial entropy atoms.
>
> ...
>
> This interpretation hinges on the non-negativity of local partial entropy atoms, however. If the local entropy atoms can be negative (as in the case of h_{cs} {ince_partial_2017}), the interpretation in terms of relative levels of surprisal is unclear. Consequently, I recommend that $h_{\min}$ or h_{sx} be used in the context of the GID.

As mentioned in the main text, the GID can return negative local partial information atoms. This is not a problem to my mind, as this just means that $h^{\mathbb{Q}}_{\partial}(\boldsymbol{\alpha}) < h^{\mathbb{P}}_{\partial}(\boldsymbol{\alpha})$: we would be *more* surprised to observe $\boldsymbol{\alpha}$ if we believed $\mathbb{P}$ than $\mathbb{Q}$. This is analgous to Finn and Lizier's argument that $i(x; y) < 0$ implies that the

"misinformation" locally dominates the informative information. However, this hinges on the local partial entropy atoms themselves being non-negative. I personally have very little intuition about what a negative local partial entropy atom might mean, and how we might interpret a negative local GID atom is the component partial entropy atoms were negative. Consequently, we recommend h_{sx} and $h_{\min}$, even though their behaviour may seem initial counter intuitive (although as I argue below, upon reflection, things like the 2-bit COPY problem are not actually problematic.)

* I have a practical concern about calculating local measures on different distributions: how to handle symbols with zero probability in one of P or Q and non-zero probability in the other (e.g. equation 18). It would be nice to see this discussed.

This is an excellent point, one that I should have considered before. I have added the following to the section defining the GID:

> The GID inherits some of the limitations of the Kullback-Leibler divergence, one of the most salient is that it is only well-defined if the support set of $\mathbb{P}(\mathbf{X})$ (which we will denote as $\mathfrak{X}^{\mathbb{P}}$) is a subset or equal to the support set of $\mathbb{Q}(\mathbf{X})$ ($\mathfrak{X}^{\mathbb{Q}}$). If there are any $\mathbf{x}$ such that $\mathbb{P}(\mathbf{x}) > 0$ and $\mathbb{Q}(\mathbf{x}) = 0$, then the ratio $\mathbb{P}(\mathbf{x})/\mathbb{Q}(\mathbf{x})$ diverges. For didactic purposes, we generally assume that $\mathfrak{X}^{\mathbb{P}} = \mathfrak{X}^{\mathbb{Q}}$, although in the case of empirical data, this may not necessarily be true. In that case, there are a few options. The simplest is to simply not use the GID: in the case where $\mathfrak{X}^{\mathbb{P}} \not\subseteq \mathfrak{X}^{\mathbb{Q}}$, then there is a sense in which the relationship between the prior and posterior is fundamentally undefined. Alternately, one could add a small amount of noise to the data, so all the probability of all $\mathbf{x} \in \mathfrak{X}^{\mathbb{Q}} > 0$. This requires assuming that $\mathbf{x}$ *can* happen under distribution $\mathbb{Q}$, but that it is so unlikely that it was not observed in a finite-sized sample. Whether this assumption is valid or not depends on the particulars of the dataset in question, and will introduce significant bias to the final computation as $h^{\mathbb{Q}}(\mathbf{x})$ will be vastly larger than $h^{\mathbb{P}}(\mathbf{x})$ and consequently, the differences will dominate the expected value. Finally, one could define a restricted support set $\mathfrak{X}^*$ such that, for all $\mathbf{x} \in \mathfrak{X}^*$, $\mathbb{Q}(\mathbf{x}) > 0$. Those $\mathbf{x} \in \mathfrak{X}^{\mathbb{P}}$ but not in $\mathfrak{X}^*$ would be excluded and the probability distribution re-normalized. This will also introduce bias, as information in $\mathbb{P}(\mathbf{x})$ will inevitably be lost when those states are thrown out. All of these strategies have drawbacks, and any prospective scientist who finds themselves in such a situation should carefully consider the trade-offs involved: to what extent are the insights that could be gained from the GID in such a case compromised by the consequences of manipulating the underlying distributions? Ultimately, the decision must be handled on a case-by-case basis.

I have taken pains to stress that, while these strategies may "work" in the sense of returning a well-defined value where there was previously undefined behaviour, they come with inherent trade-offs, and care should be taken when deciding whether to use the GID in such cases or not.

* I appreciate the bulk of the paper is a theoretical contribution which is very clear, but I think when presenting hmin and the example based on that it would be good to be more upfront about the limitations and interpretation of this measure...

We chose $h_{\min}$ largely for practical reasons (it is exceedingly easy to compute and conceptually simple), as opposed to out of any kind of commitment to it as the end-all-be-all redundant entropy function (personally, I like the h_{sx} function most, but it's somewhat more complex to introduce it). We feel like we have already addressed the point about different redundancy functions above.

* for example a €1 coin tossed in Paris, a £1 coin tossed in London a year later, and a quarter tossed in New York a year later, it is hard to see how to interpret a decomposition that says these three events share a synergistic interaction in their uncertainty.

First, one thing I should note is that this example of three coins really only makes sense in the context of the PED, not the GID. In the GID, there must be a prior and a posterior: if the prior was total independence, then in this case, the GID would return 0 redundancy and 0 synergy, just as the reviewer suggests it should. However, I do think this is still worth discussing in the context of the PED:

I am going to go out on a limb here and argue that it's actually okay to say that there is a synergistic *partial entropy* interaction here *so long as you are speaking with respect to the perspective of some observer.*

The reviewer is correct that it would be ludicrous to conclude any kind of causal dependency between the three coins. However, when considering something like the PED (or any information-theoretic measure, for that matter), we are not describing any extrinsic, causal, property of the system, but rather, we are describing how an observer observing the system would update their beliefs in response to learning partial information. When computing an information-theoretic measure from a probability distribution, the question of how that probability distribution came into the world is irrelevant. It doesn't matter if three fair coins were flipped one after another, or years apart on different planets.

This is key to our definition of PED. In prior work (`https://www.pnas.org/doi/10.1073/pnas.2300888120`), we defined the PED in terms of a PID where the "inputs" are the elements of the system and the "target" is the joint state of the whole: $I(X_1, \ldots, X_N; \{X_1, \ldots, X_N\})$. In the case of the coins, the redundant information is that information about the state of the *whole* that would be learned regardless of whether we learned the outcome of Toss 1 OR Toss 2 OR Toss 3. If we learn that Toss 1 = Heads OR Toss 2 = Heads OR Toss 3 = Heads, we can still rule out one possible joint state: (Tails, Tails, Tails).

If we think about information as the difference of a prior entropy and a posterior entropy (as in Eq. 2 in the manuscript), we can clearly see that our prior entropy is 3 bit (all joint-states are equiprobable). However, after learning that (Toss 1 = Heads) OR (Toss 2 = Heads) OR (Toss 3 = Heads) and ruling out the impossible joint state (Tails, Tails, Tails), we are left with a 7-state distribution: we have reduced our uncertainty about the joint state. Exactly how to quantify that gain depends on the specific redundancy function, of course: if one uses $h_{\min}$, we have gained 1 bit of information. If one uses h_{sx} we have gained ≈ 0.193 bit of information. Regardless of how one exactly chooses to quantify the information gained though, it seems perfectly reasonable to say that we gain some information if we learn (Toss 1 = Heads) OR (Toss 2 = Heads) OR (Toss 3 = HEADS) *regardless of when the coins were actually tossed.*

Uncertainty about the state of the whole is reduced regardless of which one we learn. Again, this is not reflecting any causal or material "integration" between the coins (there clearly is none), but rather, it describes the structure of how knowledge about the "parts" constrains the conclusions we make about the "whole" after the fact.

This setup (decomposing the information the parts disclose about the whole) is exactly the form of the classic 2-bit COPY system, and I (personally) feel like this provides a satisfactory explanation for why the 2-bit COPY behaves like it does.

The reviewer specifically mentions the synergy in the case of triple-independence. If we think of the synergistic information as that information about the target that can *only* be learned by observing all the inputs, we can see that synergy makes sense here. Suppose I learn that (Toss 1 = Heads) AND (Toss 2 = Heads). There are still two possible joint states that could be consistent with: (Heads, Heads, Tails) and (Heads, Heads, Heads). So, in the case of a random system, there is "synergistic entropy": the state of the whole can only be predicted if we know the states of all the parts.

* Abstract: "in as a" $<->$ "as a"

Fixed

* P4, Para "The local entropy h(X)", should be lower case X?

Fixed

* P5, after eq 11. "sub" -¿ "sum"

Fixed

* P7, last para, If X1 = 0 and X2 = 0 (and lines below). Might be getting confused with notation, but should these not be lower case?

I think this is a case where the notation is inherently ambiguous. Typically I denote specific states of random variables as $X_1 = x_1$. So if the state in question is 0, then you could write $X_1 = x_1 = 0$.

* It would be nice to see some citations to earlier practical applications of PID in neuroimaging e.g. 10.1371/journal.pbio.2006558, 10.1016/j.cub.2019.04.067 (this one actually uses PID in 3 different ways, to justify use of single sources, for comparison of encoding models, and for comparison of decoding from the predictions of encoding models).

I have added these to the references, however, given the theoretical and generally domain-agnostic nature

of this paper, I think a detailed discussion of these papers is outside of it's scope. Giving them the treatment they deserve would require introducing a lot of neuroscience-specific material which I think would muddy the basic story.

Reviewer 2

* Synergy interpretation in TSE complexity.

I have expanded the introduction of the TSE complexity a little bit, and tried to stress that the integration/segregation interpretation is motivated by the behaviour of the TSE function itself (the so-called "TSE leaf plot")

> Formally, the TSE is highest when, on average, subsets of a given system are statistically independent (i.e. the total correlation is zero), but the whole system itself strongly deviates from independence (i.e. the total correlation of the whole is high). This suggests a natural link to synergy: deviation from independence in the whole, but none of the parts at any scale. In the original presentation of the TSE complexity, Tononi, Sporns, and Edelman showed that the measure has a characteristic, inverted-U shape: when all elements are independent (global segregation), the complexity is low, and similarly, when all elements are synchronized (global integration), the complexity is similarly low. Complexity is highest in an interstitial zone combining integration and segregation.

* The GID of $I(X_1, X_2 : Y)$.

I would like to thank the reviewer for their keen eye on this one. I have fixed Eq. 26 so that it is the expected D_{KL}:

$$I(X_1, X_2; Y) := \mathbb{E}_{\mathbb{P}(Y)}[D(\mathbb{P}(X_1, X_2|Y = y)||\mathbb{P}(X_1, X_2))] \tag{1}$$

However, I do not think this resolves the discrepancy between the number of GID atoms. For each possible state of $Y = y$, we have a D_{KL} of two variables: the prior ($\mathbb{P}(X_1, X_2)$, which is always the same), and the posterior, $\mathbb{P}(X_1, X_2|Y = y)$, which is different for each value y. The GID of each of those D_{KL}s produces four atoms (the typical set of a redundant, synergistic, and two unique atoms). When we then compute the expected value of each of those atoms w.r.t. to $\mathbb{P}(Y)$, we still end up with just four atoms: the expected redundancy, synergy, and unique information terms. It is not clear to me where the "higher order" atoms (like $\{X_1, X_2\}\{X_1, Y\}\{X_2, Y\}$) might come from in this setup.

To verify the intuition, I used the `SxPID` package to do the GID for the logical-AND gate (equivalent to using the h_{sx} function), and can verify that it does recover the usual four-atom PID. At the end of this document you can see the Python code.

If I had to take a guess as to why there is this discrepancy (four atoms versus eighteen atoms), it would be that it reflects a fundamental difference between the perspectives inherent in each formulation of the mutual information. In the "canonical" formulation of the mutual information as a Kullback-Leibler divergence, the analysis implicitly treats the system as a triad of three interacting elements:

$$I(X_1, X_2; Y) = D(\mathbb{P}(X_1, X_2, Y)||\mathbb{P}(X_1, X_2) \times \mathbb{P}(Y)) \tag{2}$$

There is, to my mind, a sense in which this is naturally understood as saying something about a three-dimensional probability distribution.

In contrast, in the "conditional" case, (see the now-corrected Eq. 26 in the main text), the natural understanding is of a two element distribution (X_1, and X_2), the structure of which is modulated by the "external" variable Y. Even though both formulations are mathematically equivalent and inter-convertable, there may be some sense in which they *are* different functions: whether the target Y is considered part of the "system", or whether it is external to the "system." Perhaps this difference in formalizations is just something that the field has not ever had to consider deeply and so has gone unnoticed (or at least, I have never considered it until now).

If this is true, then the discrepancy between the 18-atom case and the four-atom case is actually reflecting a meaningful difference in how the experimenter/analyst chooses to think about the mutual information.

I have added the following expanded discussion to the manuscript:

> How can we make sense of this unusual feature? One possible explanation is that, while both formulations (Eqs. 26 and 30) are the same mutual information, they implicitly privilege different ways of thinking about the relationship between X_1, X_2, and T. In the first case (Eq. 26), the system under study is naturally understood as the two-dimensional pair of X_1 and X_2: the target variable T is "external" in some sense and modulates the behaviour of X_1 and X_2 from outside the system. In contrast, in the case of Eq. 30, the natural perspective is of a three-element system X_1, X_2, and T, which are decomposed together, producing eighteen atoms. Currently, this is merely a hypothesis that attempts to explain this discrepency, but it suggests that the perspectives or biases of the observer/analyst deploying an information-theoretic tool can inform on the apparent structure of the system under study.

I understand that this sounds somewhat far-fetched. However, having beat my head against the problem for a few weeks, and empirically verifying it with code, I am pretty convinced that this a "real" distinction that the GID is picking up on. I am, of course, open to being proved wrong if there's some subtle (or blindingly obvious) error I have made somewhere.

* The author could add for clarity that $X \perp Y$ stands for X and Y are statistically independent. Since it is reoccurring throughout the manuscript with no introduction.
We have added an introduction of the $\perp$ symbol.

* This needs a citation.
We have added the following to the manuscript elaborating on the problem of picking a redundancy function and citing Artemy Kolchinsky's recent paper that directly tests a variety of redundancy measures:

> "Since the original introduction of the PID, various teams have proposed a plethora of redundancy functions that satisfy the Williams and Beer axioms and consequently induce the redundancy lattice. These different redundancy functions can return very different results making the problem of picking the "right" function a tricky one. For a partial review, see {kolchinsky_novel_2022}."

* Rather than writing Informtion which is read as the product of variable I, n, f , and so on. The author can use the command "mathrm" so that information is an operator and appears as such Information. The same applies to prior and posterior in the same equation.
Fixed, this is a macro I am pleased to learn about.

* This needs a citation too
I have added a citation to W&B here.

* This page could benefit from rewriting. We also suggest adding a figure for the redundancy lattice. Especially since the author refers later to the top half and the bottom half of the redundancy lattice without defining them (they are only clear for Information decomposition experts). This for completeness could be cleared in one figure.
I have added a figure with the following caption:

> **The partial information lattice for three, interacting elements.** For three elements X_1, X_2, and X_3, the set of all sources is organized into a partially-ordered lattice structure. At the bottom of the lattice is the triple redundancy: $\{X_1\}\{X_2\}\{X_3\}$, which is the information that can be learned by observing X_1 alone or X_2 alone or X_3 alone. At the top is the triple synergy: $\{X_1, X_2, X_3\}$, which is the information can can only be learned by observing X_1, X_2, and X_3 together. The lattice represents a transition from redundancy-dominated interactions at the bottom (highlighted in red) towards synergy-dominated interactions at the top (highlighted in blue). In the middle are the three unique information atoms, corresponding to X_1 alone, X_2 alone, and X_3 alone.

I have also re-written the explanation of the XOR gate decomposition to make it more parsimonious.
* There is a missing parenthesis in the term

Fixed.

* This sentence could be made clearer.
I have rephrased it to make it more concise.

* Also, if we are not mistake their notation...
This was a typo on my part - fixed.

* We think $\mathbb{Q}$ is our prior, no?
Also a typo - fixed

* Is it more common to write
Fixed

* The author is using...also used in Table 1.
Fixed

* Additionally, this paragraph needs rewriting. Possibly dividing it into steps so that the reader can grasp it more easily.
I have attempted to refine this paragraph and make each step unambiguous and motivated.

* The author could elaborate that the synergy term is $\{1, 2, 3\}$ and also give examples of the redundancy terms.
I'm afraid I'm not sure what the authors mean by this.

* The author uses the term disintegration to refer to complete segregation...This is confusing to the reader.
I have swapped out "disintegrated" with "segregated" for consistency.

* This section could benefit from a division into subsections with meaningful titles
This section has been re-worked: I've added section titles, tried to refine some of the arguments and add more literature. I hope that it reads more smoothly.

* The author talks about the potential impact of the work on cognitive science
I understand the point the reviewers are making, and I have expanded this section to make my argument a little clearer and less ambiguous. With respect to the specific critique about semantic information - I am willing to cross that bridge if or when I come to it. I think, as it stands, the point is worth making despite it's limitations.

* Integrated infomation decomposition ΦID...
I am very much interested in this line of work (and if the reviewers are interested, I absolutely would like to talk this through in more detail post-publication), however, I think a more detailed exploration of the ΦID would be too much for this paper. I have already introduced a fair amount of mathematical machinery, and I think that adding in the double-redundancy lattice would be too much. However, as a longer-term project, I would love to collaborate on a kind of "grand unified theory" of information decomposition that combined PID, PED, GID, and ΦID into a coherent framework. Once again, if the reviewers would be interested in such a thing, I would absolutely invite them to reach out for further discussion.

I have played around a bit with attempting to "squish" the 166 GID atoms into the sixteen ΦID atoms, although without much success. Part of the issue is that the ΦID atoms are sensitive to the order of time when the GID atoms aren't. Since the GID and the ΦID should sum to the same value (the mutual information), exactly how to map the different classes of atoms on each-other stumped me.

```python
#!/usr/bin/env python3
# -*- coding: utf-8 -*-
"""
Created on Sun Nov 12 20:56:05 2023

@author: thosvarley
"""

# Using h_sx b/c the package does it all for me.
from sxpid import SxPID

# Probability distribution for the logical-AND
p_and = {
(0,0,0) : 1/4,
(0,1,0) : 1/4,
(1,0,0) : 1/4,
(1,1,1) : 1/4,
}

# PID for the logical-AND.
_, avg = SxPID.pid(p_and)

# I(X_1, X_2 ; Y) = sum(PI atoms)
# This will get referenced later.
mi = sum([avg[key][-1] for key in avg.keys()])

# Doing the local PED for the prior (maximum entropy of X_1, X_2)
prior = {
(0,0,"A") : 1/4,
(0,1,"B") : 1/4,
(1,0,"C") : 1/4,
(1,1,"D") : 1/4
}

# Local PED for the prior
ptw_prior, _ = SxPID.pid(prior)

# Posterior when y = 0
posterior_y0 = {
(0,0,"A") : 1/3,
(0,1,"B") : 1/3,
(1,0,"C") : 1/3,
(1,1,"D") : 0
}

# Posterior when y = 1
posterior_y1 = {
(0,0,"A") : 0,
(0,1,"B") : 0,
(1,0,"C") : 0,
(1,1,"D") : 1
}
```

```
# Local PEDs for the posteriors
ptw_posterior_y0, _ = SxPID.pid(posterior_y0)
ptw_posterior_y1, _ = SxPID.pid(posterior_y1)

# The four atoms
atoms = {((1,),(2,)), ((1,),), ((2,),), ((1,2),)}

# Doing the DKL when y = 0
dkl_y0 = {atom : sum(
[posterior_y0[state]*(ptw_prior[state][atom][0] - ptw_posterior_y0[state][atom][0])
for state in ptw_posterior_y0.keys()])
for atom in atoms}

# Doing the DKL when y = 1
dkl_y1 = {atom : sum(
[posterior_y1[state]*(ptw_prior[state][atom][0] - ptw_posterior_y1[state][atom][0])
for state in ptw_posterior_y1.keys()])
for atom in atoms}

# Parial local w.r.t to Y.
exp_y = {atom : (3/4*dkl_y0[atom]) + (1/4*dkl_y1[atom]) for atom in atoms}

# E_{Y}[ DKL( P(X_1, X_2 | Y = y) || P(X_1, X_2)) ]
sum_atoms = sum(exp_y.values())

print(" ")
print(sum_atoms == mi)
```
